# Effects of a blend of *Saccharomyces cerevisiae*-based direct-fed microbial and fermentation products on plasma carbonyl-metabolome and fecal bacterial community of beef steers

**DOI:** 10.1186/s40104-019-0419-5

**Published:** 2020-02-17

**Authors:** James A. Adeyemi, Sunday O. Peters, Marcos De Donato, Andres Pech Cervantes, Ibukun M. Ogunade

**Affiliations:** 1 College of Agriculture, Communities, and the Environment, Kentucky State University, Frankfort, KY 40601 USA; 2 Department of Animal Science, Berry College, Mount Berry, GA 30149 USA; 3 Tecnologico de Monterrey, Escuela de Ingenieria y Ciencias, Queretaro, Mexico; 4 Agricultural Research Station, Fort Valley State University, Fort Valley, GA 31030 USA

**Keywords:** Beef steer, Carbonyl, Fecal bacteria, Lactate-utilizing bacteria, Plasma metabolomics

## Abstract

**Background:**

Previous studies have evaluated the metabolic status of animals fed direct-fed microbial (DFM) using enzyme-based assays which are time-consuming and limited to a few metabolites. In addition, little emphasis has been placed on investigating the effects of DFM on hindgut microbiota. We examined the effects of dietary supplementation of a blend of *Saccharomyces cerevisiae*-based DFM and fermentation products on the plasma concentrations of carbonyl-containing metabolites via a metabolomics approach, and fecal bacterial community, via 16S rRNA gene sequencing, of beef steers during a 42-day receiving period. Forty newly weaned steers were randomly assigned to receive a basal diet with no additive (CON; *n* = 20) or a basal diet supplemented with 19 g of Commence™ (PROB; *n* = 20) for a 42-day period. Commence™ (PMI, Arden Hills, MN) is a blend of 6.2 *×* 10^11^ cfu/g of *S. cerevisiae*, 3.5 × 10^10^ cfu/g of a mixture of *Enterococcus lactis, Bacillus subtilis, Enterococcus faecium*, and *Lactobacillus casei*, and the fermentation products of these aforementioned microorganisms and those of *Aspergillus oryzae* and *Aspergillus niger*. On d 0 and 40, rectal fecal samples were collected randomly from 10 steers from each treatment group. On d 42, blood was collected for plasma preparation.

**Results:**

A total number of 812 plasma metabolites were detected. Up to 305 metabolites [fold change (FC) ≥ 1.5, FDR ≤ 0.01] including glucose, hippuric acid, and 5-hydroxykynurenamine were increased by PROB supplementation, whereas 199 metabolites (FC ≤ 0.63, FDR ≤ 0.01) including acetoacetate were reduced. Supplementation of PROB increased (*P* ≤ 0.05) the relative abundance of *Prevotellaceae* UCG-003, *Megasphaera*, *Dorea*, *Acetitomaculum,* and *Blautia*. In contrast, the relative abundance of *Elusimicrobium*, *Moheibacter*, *Stenotrophomonas, Comamonas,* and uncultured bacterium belonging to family p-2534-18B5 gut group (phylum Bacteroidetes) were reduced (*P* ≤ 0.05).

**Conclusions:**

The results of this study demonstrated that supplementation of PROB altered both the plasma carbonyl metabolome towards increased glucose concentration suggesting an improved energy status, and fecal bacterial community, suggesting an increased hindgut fermentation of the beef steers.

## Background

Direct-fed microbials (DFM) are commonly used in livestock production systems to improve the metabolic and energy status of animals especially during stress periods, thereby leading to improved animal productivity [1–3]. Several studies have suggested that the effects of DFM on the metabolic and energy status of ruminants are attributed to the modulation of the rumen microbiota, improved gut integrity, and increased intestinal nutrient absorption [4–6]. The majority of studies using DFM have been targeted toward enhancing metabolism of lactic acid to propionate through inoculation of *Saccharomyces cerevisiae* and lactate-utilizing bacteria [1, 2]. Other studies have evaluated the approach of using lactic acid-producing bacteria, such as *Lactobacillus* and *Enterococcus* spp., to enhance production of lactic acid which can be metabolized to propionate by ruminal lactate-utilizing bacteria [3]. In recent years, most commercial DFM products are formulated to contain a mixture of these aforementioned micro-organisms and their fermentation products in order to ensure efficacies and multi-factorial response.

Previous studies have evaluated the metabolic status of animals fed supplemental DFM using enzyme-based assays for individually determining certain metabolites, such as blood glucose and beta-hydroxybutyric acid [3, 7]; however, these assays are time-consuming and limited to a few metabolites. Recent advances in metabolomics have provided the opportunity to simultaneously quantify the relative concentrations of multiple metabolites [8]. Metabolites containing a carbonyl group are important classes of molecules including ketones and aldehydes, such as steroids and sugars [9]. These metabolites are common intermediates of energy metabolism; therefore, their concentrations in the blood can reflect the energy status of animals. The use of high-performance chemical isotope labeling (CIL) liquid chromatography mass spectrometry **(**LC-MS**)-**based metabolomics has provided the opportunity to analyze the carbonyl submetabolome of biofluid with high coverage, accuracy, and precision [9]. Analysis of plasma carbonyl-metabolome of animals will provide an opportunity to understand better how dietary supplementation of DFM affects animal performance and health.

In our companion study [10], supplementation of a blend of *S. cerevisiae*-based DFM and fermentation products (PROB) improved the performance and health of newly-weaned beef steers which might be attributed to improved energy status of the animals. Therefore, we hypothesized that supplementation of PROB would alter the plasma carbonyl submetabolome due to its positive effects on the performance of the steers. The objective of this study was to apply a CIL/LC-MS-based quantitative untargeted metabolomics to evaluate the effects of PROB on the plasma concentrations of carbonyl-containing metabolites in beef steer.

Also in our companion study [10], there was a tendency for a lower fecal pH in steers fed PROB diet, which is possibly an indication of increased volatile fatty acids due to increased hindgut fermentation and altered hindgut microbiota. Little emphasis has been placed on investigating the effects of DFM on hindgut microbiota. The fecal pH and bacterial composition can particularly reflect the true condition of the hindgut microbiota [11]. Therefore, the second objective of this study was to evaluate the effects of PROB supplementation on fecal bacterial community of beef steers.

## Methods

The Kentucky State University Animal Care and Use Committee approved all procedures and protocols for this experiment.

### Animals and feeding

Forty newly-weaned Angus crossbred steer calves [7 d post-weaning; 210 ± 12 kg of body weight (BW); 180 ± 17 d of age] were stratified by BW into 4 weight blocks. The steers were randomly assigned (within each weight block) to 1 of 2 treatments and housed in individual slatted floor pens (1 steer per pen). These calves were from a single source and were not transported. The treatments were a basal diet with no additive (CON; *n* = 20) or a basal diet supplemented with 19 g/d of Commence™ (PROB; *n* = 20) for a 42-day period. Commence™ (PMI, Arden Hills, MN) contains a blend of 6.2 *×* 10^11^ cfu/g of *S. cerevisiae*, 3.5 × 10^10^ cfu/g of a mixture of *Enterococcus lactis, Bacillus subtilis, Enterococcus faecium, and Lactobacillus casei*, and the fermentation products of these aforementioned microorganisms as well as those of *Aspergillus oryzae* and *Aspergillus niger*. The basal diet was fed daily as a total mixed ration (TMR) at 08:00 h (See Additional file 1: Table S1). The additive was top-dressed daily on the TMR in the form of a premix using dried distillers grains with solubles for the PROB treatment while a similar premix with no additive was top-dressed for the CON treatment.

### Feces and blood collection

On d 0 and 40, rectal fecal samples were collected randomly from 20 steers (10 steers from each treatment) approximately 4 h after feeding and immediately stored at − 80 °C until further analysis. On d 42, 15 mL of blood was taken before the morning feeding. The blood was collected from the coccygeal vessels into tubes containing sodium heparin (ThermoFisher Scientific, Wilmington, DE) for preparation of plasma and immediately stored at − 80 °C until further analysis.

### Analysis of carbonyl-containing metabolites

Relative quantification of metabolites containing a carbonyl group (carbonyl-metabolome) in plasma samples collected on d 42 was done using a CIL/LC-MS method. This method uses a differential ^12^C- and ^13^C- dansylhydrazine (DnsHz) labeling to change the chemical and physical properties of carbonyl metabolites to enable them to be efficiently separated by LC and ionized by electrospray ionization MS [9]. The workflow of the analysis as well as details of dansylhydrazine labeling, LC-UV normalization, and LC-QTOF-MS setup and running conditions have been previously reported [9]. Briefly, 90 μL of LC-MS grade methanol was mixed with 30 μL of plasma sample for protein precipitation. The methanol extract was then dried after incubation at − 20 °C for 2 h and then re-dissolved in 30 μL of LC-MS grade water. For labeling, 30 μL of pH-adjusting reagent and 30 μL of ^12^C_2_-labeling was mixed with 20 μL of the individual plasma samples and the pooled sample (prepared by mixing 70 μL of each plasma sample). In addition, 30 μL of pH adjusting reagent and 30 μL of ^13^C_2_-labeling was mixed with another 20 μL of pooled sample. After vortexing and spinning down (using a SpeedVac to remove the acid catalyst), the mixture was incubated at 40 °C for 60 min. Then the mixture was cooled down in − 80 °C freezer for 10 min to stop the labeling reaction. The mixture was then dried using a centrifugal vacuum concentrator. Finally, the dried mixture containing the labelled metabolites was re-dissolved in 100 μL of acetonitrile/Water (50:50, v/v). The ^12^C_2_-labeled individual sample was mixed with ^13^C_2_-labeled reference sample in equal amount according to carbonyl-labeling LC-UV normalization results. The solution was diluted and centrifuged at 15294×*g* for 10 min before injecting into Bruker Elute LC linked to Bruker Impact II QTOF MS for analysis. Quality control (QC) sample (prepared by mixing equal amount of a ^12^C-labeled and a ^13^C-labeled pooled sample) was injected every 10 sample runs to monitor instrument performance.

### Fecal DNA extraction, sequencing, and diversity analysis

Total DNA was extracted from the feces with a PowerSoil DNA Isolation Kit (MOBIO Laboratories Inc.; Carlsbad, CA, USA). The integrity of the DNA was verified using 0.8% agarose gel electrophoresis. DNA concentration was determined using a NanoDrop 3300 (ThermoFisher Scientific, Wilmington, DE). The DNA samples were prepared for sequencing according to the Illumina 16S Metagenomic Sequencing Library protocols to amplify the V3–V4 region. Amplification of the genomic DNA (10 ng) was performed using polymerase chain reaction (PCR) conditions: 94 °C for 3 min followed by 35 cycles of 94 °C for 15 s, 55 °C for 45 s, and 72 °C for 1 min, followed by a final elongation step of 8 min at 72 °C. 519F:5´-CCTACGGGNGGCWGCAG-3´ and 806R: 5´-GACTACHVGGGTATCTAATCC-3´ were used as the primer sequences. Quantification of the final purified product was done according to qPCR Quantification Protocol Guide (KAPA Library Quantification kits for Illumina Sequencing platforms, Wilminton, MA, USA), and the quality was checked using the LabChip GX HT DNA High Sensitivity Kit (PerkinElmer; Waltham, MA, USA). Sequencing was performed on a paired-end Illumina MiSeq platform to generate 300-bp paired-end raw reads. Raw paired end reads were joined using FLASH2 v.c41a82e [12]. The merged reads were quality-filtered to remove adaptors and low quality bases using filtering conditions of Trimmomatic v0.33 [13]. The resulting trimmed reads were compared with the reference database (the “Gold” database, [14]) using the UCHIME algorithm [15] to detect and remove the chimeric sequences. After processing and quality filtering, UCLUST in QIIME (version 1.8.0 [15];) was used to cluster the tags with 97% similarity to acquire the Operational Taxonomic Units (OTU). The representative OTU sequences were annotated using the rRNA database (Silva) for taxonomic assignment. Shannon index, a measure of alpha diversity, and unweighted unifrac distance, a measure of beta diversity were generated using the QIIME software package with a script core_diversity_analyses.py [16]. The datasets in this study are available in the NCBI BioProject database with the SRA accession number: PRJNA588450.

### Data and statistical analysis

Variables such as relative abundance of fecal bacteria and diversity indices were analyzed as a randomized complete block design using the GLIMMIX procedure of SAS 9.4 (SAS Inst. Inc., Cary, NC). The model included the fixed effects of treatment, random effects of block (BW as the block). Day 0 data were used as covariates. Each steer housed in an individual pen served as the experimental unit. Linear discriminant analysis effect size (LEfSe), which uses a Kruskal-Wallis (KW) test and the logarithmic linear discriminant analysis (LDA) score, was used to identify the most differentially abundant taxa [17]. The significance estimate for the KW test and the LDA score cutoff were 0.05 and 4.0, respectively.

For the metabolomics data, 48 LC-MS data (4 blank group samples, 4 QC samples, 20 CON samples, and 20 PROB samples) were first exported to .csv file with Bruker DataAnalysis 4.4. The exported data were uploaded to IsoMS Pro 1.0.10. After format conversion, data quality check and data processing were performed according to procedures described by Mung and Li [18]. Metabolite identification was performed using a three-tier identification approach. In tier 1, peak pairs were searched against the CIL Library [DnsHz (ketone and aldehyde)] based on accurate mass and retention time [19]. In tier 2, high-confidence identification based on accurate mass and predicted retention time matches was performed using linked identity library (LI Library) [20]. In tier 3, the remaining peak pairs were searched, based on accurate mass match, against the MyCompoundID (MCID) library composed of 8,021 known human endogenous metabolites (zero-reaction library) and their predicted metabolic products from one metabolic reaction (one-reaction library) and two metabolic reactions (two-reaction library) [20]. The mass accuracy tolerance window was set at 10 ppm for all searches, and the retention time tolerance window was set to 30 s for CIL library and 200 s for LI library.

Partial least squares discriminant analysis (PLS-DA) scores plot was used to visually show the differences between plasma metabolome of CON and PROB. Univariate (volcano plot) was generated using IsoMS Pro 1.0 [18] by plotting the fold change (FC; PROB/CON) against the false discovery rate (FDR) adjusted *P*-value. Relative concentrations of metabolites with FC ≥ 1.5 or ≤ 0.67 having FDR ≤ 0.01 were differentially increased or decreased relative to CON, respectively. Pearson correlation analysis was used to examine the association between the relative concentrations of the identified plasma metabolites and growth performance indices (feed efficiency (FE) and average daily gain (ADG). The Pearson correlation coefficients were generated using R software (http://www.r-project.org). Pearson correlations were declared significant at *P* ≤ 0.10.

## Results

The effects of PROB supplementation on performance and health of the beef steers were reported in our previous study [10]. Briefly, compared with CON, dietary supplementation of PROB increased final body weight (270 vs. 260 kg; *P* = 0.01; SE = 2.67) and ADG (1.42 vs. 1.23 kg; *P* = 0.04; SE = 0.06). There was a tendency for improved FE with PROB supplementation (0.232 vs 0.209; *P* = 0.10; SE = 0.01). There was a tendency for lower fecal pH (*P* = 0.08; 6.46 vs. 6.62; SE = 0.06) in steers fed PROB diet.

### Effects of PROB on plasma carbonyl-containing metabolites

**Fig. 1:**
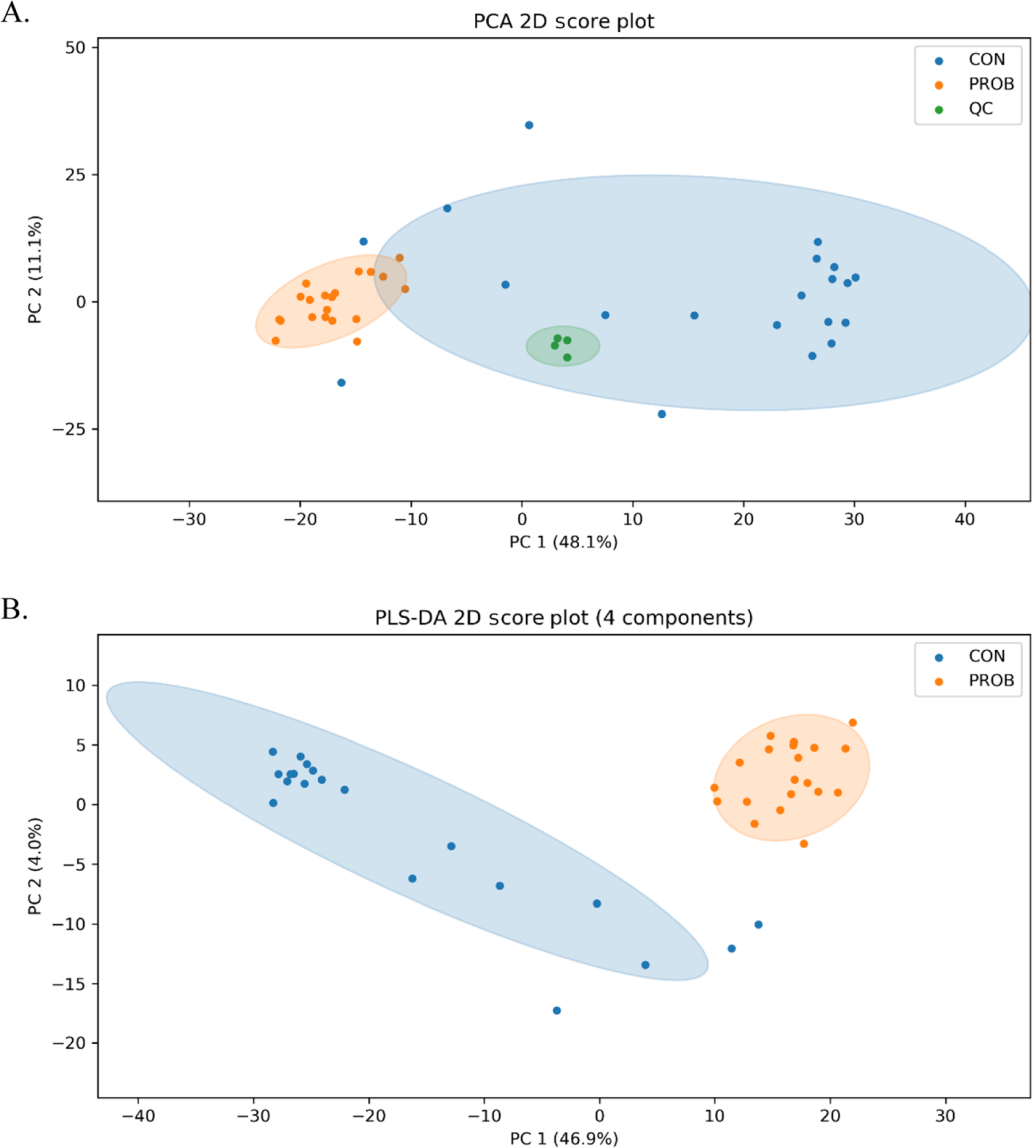
**a** Principle component analysis (PCA) scores plot (with quality control), **b** Partial least squares discriminant analysis (PLS-DA) scores plot of the two treatments. CON = control; PROB = a blend of *Saccharomyces cerevisiae*-based direct-fed microbial and fermentation products fed at 19 g/steer per day

The mass-to-charge ratio (m/z) of 251.0849 was used as a background peak to check the mass accuracy of all the samples as well as 4 QC and 4 blank group samples. The appearance and mass of the peak was consistent for all runs and all scanned m/z were within the expected range, showing good stability and mass accuracy for the data acquisition (See Additional file 2: Figure S1). Approximately 757 ± 44 peak pairs per run were detected (See Additional file 3: Table S2) and a total number of 812 unique peak pairs were detected (See Additional file 4). Fourteen peak pairs were positively identified in tier 1, 29 peak pairs were putatively identified with high confidence in tier 2 while 104, 434 and 661 peak pairs were matched in the zero-, one- and two-reaction libraries, respectively in tier 3. Thus, 704 pairs (86.7% of 812 unique pairs) were positively identified or putatively matched. Both PCA and PLS-DA plots showed clear separations between the CON and PROB samples, indicating that the plasma carbonyl-containing metabolome of the beef steers was altered by PROB supplementation (Fig. 1). The permutation test result (*P* = 0.01) and PLS-DA cross validation results (*R*^*2*^ = 0.988, Q^2^ = 0.788) confirm the validity of the PLS-DA model (See Additional file 5: Figure S2).

**Fig. 2:**
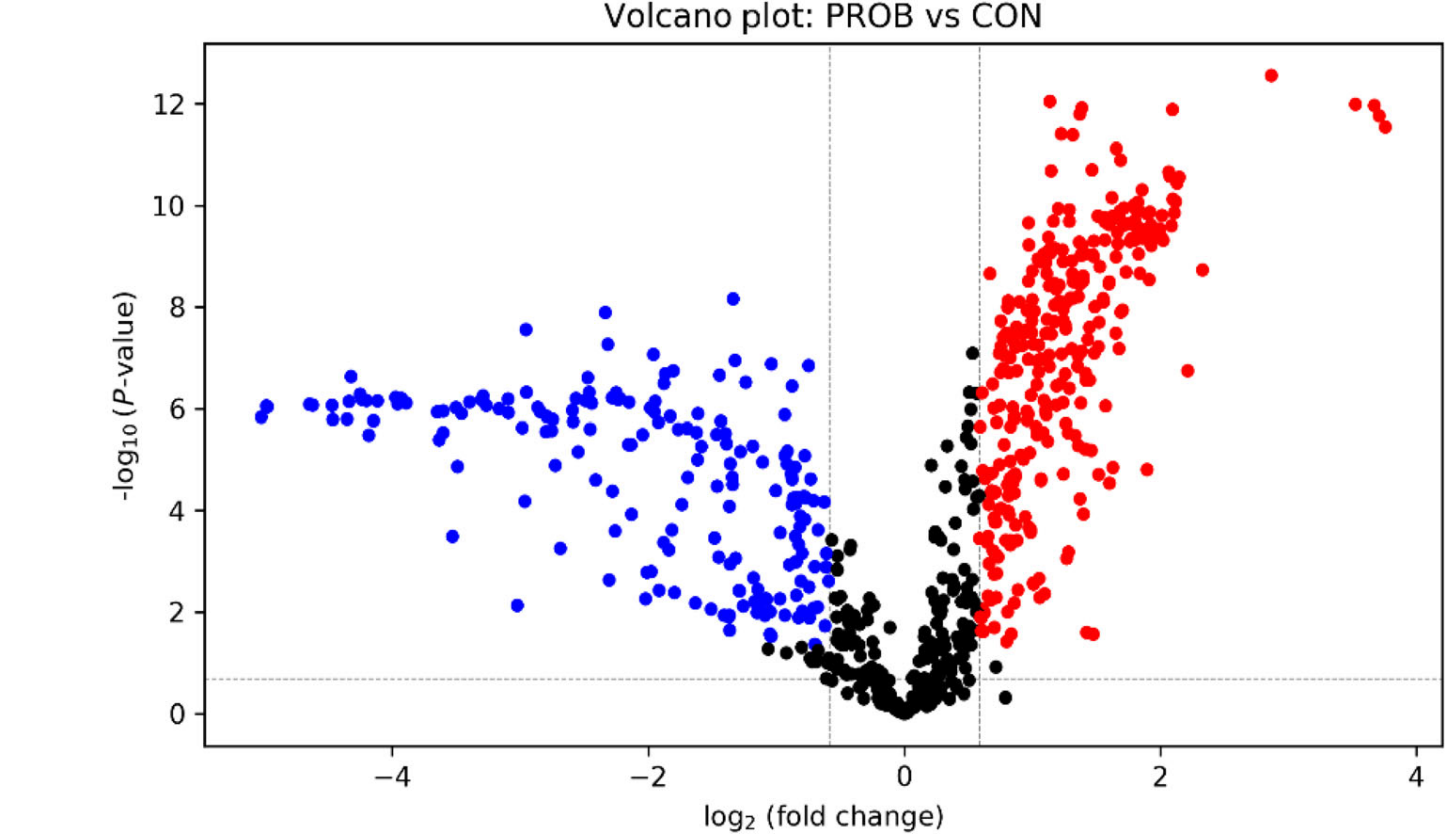
Volcano plot showing the differential metabolites. FC > 1.5, FDR ≤ 0.05 (in red): significantly increased relative to CON. FC < 0.67, FDR ≤ 0.05 (in blue): significantly reduced relative to CON. CON = control; PROB = a blend of *Saccharomyces cerevisiae*-based direct-fed microbial and fermentation products fed at 19 g/steer per day

Volcano plot analysis showed that 305 metabolites (FC ≥ 1.5, FDR ≤ 0.01, in red) were increased by PROB supplementation, whereas 199 metabolites (FC ≤ 0.63, FDR ≤ 0.01, in blue) were reduced (Fig. 2). Among those that were differentially altered, 9 metabolites were positively identified in tier 1, 17 metabolites were putatively identified with high confidence in tier 2, and 413 metabolites were matched in tier 3 (See Additional file 6). The differentially altered (FC ≥ 1.5 or ≤ 0.67, FDR ≤ 0.01) metabolites that were positively and putatively identified with high confidence are shown in Table 1. Relative to CON, 23 metabolites including glucose, fructose, galactose, glyceraldehyde, hippuric acid, glycoaldehyde, and 5-hydroxykynurenamine were increased (FC ≥ 1.5, FDR ≤ 0.01) in steers fed PROB diet. In contrast, 3 metabolites (lactose, acetoacetate, and 2-dehydro-3-deoxy-L-arabinonate) were decreased (FC ≤ 0.63, FDR ≤ 0.01).

**Table 1:** Identified peak pairs (tier 1 and tier 2) that were affected by dietary supplementation of a blend of *S. cerevisiae*-based direct-fed microbials and fermentation products

Compound	Fold change	FDR
Galactose	2.60	< 0.01
Lactose	0.46	< 0.01
Glucose	2.62	< 0.01
Fructose	2.31	< 0.01
Isomer of fructose	2.30	< 0.01
Isomer of glyceraldehyde	2.01	0.01
Glyceraldehyde	2.01	0.01
Hippuric acid	2.13	< 0.01
Phenylacetylglycine	1.98	0.01
5-Hydroxykynurenamine	2.63	< 0.01
4-Oxoglutaramate	1.82	< 0.01
2-Dehydro-3-deoxy-*D*-glucarate	1.80	< 0.01
3-Fumarylpyruvate	2.58	< 0.01
1-Deoxy-*D*-xylulose 5-phosphate	2.36	< 0.01
Glycolaldehyde	1.63	0.01
Hydroxypyruvate	1.60	< 0.01
2-dehydro-3-deoxy-*L*-arabinonate	0.30	< 0.01
Acetoacetate	0.62	0.01
Dehydroascorbate - 2 tags	1.74	0.01
3-Methylindolepyruvate	3.72	< 0.01
(S)-2-Aceto-2-hydroxybutanoate	2.96	< 0.01
5-Oxopentanoate	2.30	< 0.01
(R)-3-Hydroxy-3-methyl-2-oxopentanoate	2.96	< 0.01
2-Dehydropantoate	3.28	< 0.01
Isomer of (S)-2-aceto-2-hydroxybutanoate	3.51	< 0.01
Isomer of (S)-3-methyl-2-oxopentanoic acid	2.19	< 0.01

FC: fold change relative to control

*P*-value was calculated from student’s *t*-test

Tier 1 - Positive identification (chemical isotope labelling library);

Tier 2 - High confidence putative identification (linked identity library library)

Only metabolites with both fold-change ≥1.5 or ≤ 0.67 and FDR ≤ 0.01 are shown

Plasma concentrations of 5-oxopentanoate, isomer of (S)-2-aceto-2-hydroxybutanoate, and isomer of (S)-3-methyl-2-oxopentanoic acid were positively correlated with both FE and ADG while relative concentrations of 3-(4-hydroxyphenyl)pyruvate, (S)-2-aceto-2-hydroxybutanoate, (R)-3-hydroxy-3-methyl-2-oxopentanoate, and 2-dehydropantoate were positively correlated with ADG (Table 2).

**Table 2:** Pearson correlations between plasma metabolites and performance indices of the beef steers

	ADG		FE	
	r	*P*-value	r	*P*-value
3-(4-Hydroxyphenyl)pyruvate	0.27	0.09	0.22	0.18
(S)-2-Aceto-2-hydroxybutanoate	0.31	0.06	0.25	0.13
5-Oxopentanoate	0.43	0.01	0.36	0.03
(R)-3-Hydroxy-3-methyl-2-oxopentanoate	0.31	0.06	0.24	0.14
2-Dehydropantoate	0.31	0.06	0.25	0.12
Isomer of (S)-2-aceto-2-hydroxybutanoate	0.33	0.04	0.27	0.09
Isomer of (S)-3-methyl-2-oxopentanoic acid	0.32	0.05	0.30	0.07

Only metabolites with correlation coefficient (r) of *P*-value ≤0.10 for either average daily gain (ADG) or feed efficiency (FE) are shown

### Effects of PROB on fecal bacterial community

After quality filtering and the removal of chimeric sequences, a total number of 1,408,962 sequences were produced (an average of 70,448 sequences per sample). The mean read length of quality checked and merged reads was 451 bp.

**Fig. 3:**
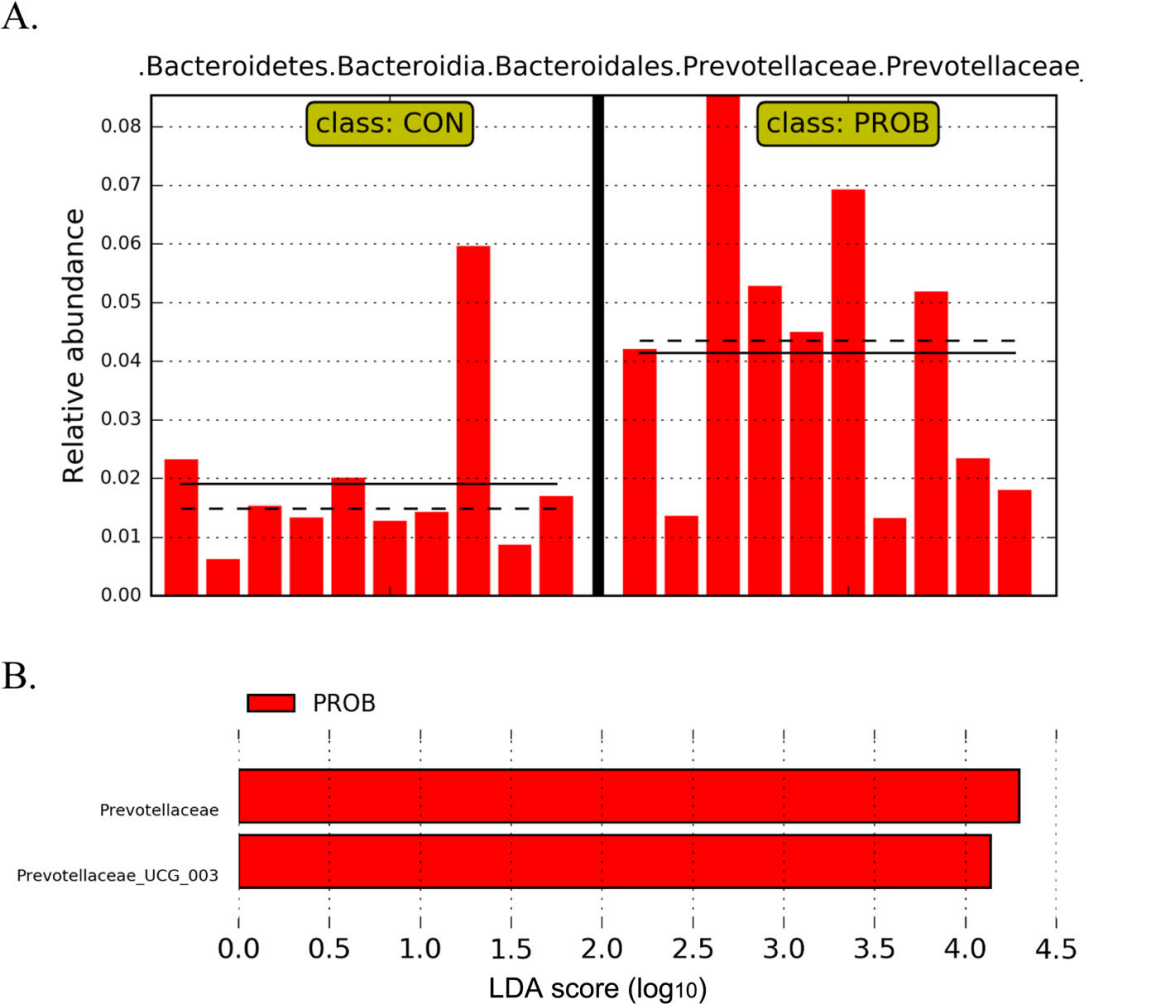
**a**. Relative abundance of *Prevotellaceae* UCG-003 and **b**. Linear discriminant analysis effect size (LEfSe) of fecal bacterial population of beef steer fed no (CON) or 19 g/d of a blend of *S. cerevisiae*-based direct-fed microbials and fermentation products (PROB; PMI, Arden Hills, MN, USA). Linear discriminant analysis effect size plot shows the most differentially abundant taxa according to the logarithmic linear discriminant analysis (LDA) ≥ 4.0

There were no treatment effects (*P* > 0.10) on alpha (Shannon index) and beta diversity (unweighted unifrac distance) (See Additional file 7: Figure S3). Supplementation of PROB increased (*P* ≤ 0.05) the relative abundance of *Prevotellaceae* UCG-003, *Megasphaera*, *Dorea*, *Acetitomaculum,* and *Blautia*. In contrast, the relative abundance of *Elusimicrobium*, *Moheibacter*, *Stenotrophomonas, Comamonas,* and uncultured bacterium belonging to family p-2534-18B5 gut group were reduced (*P* ≤ 0.05) (Table 3). Using LEfSe, *Prevotellaceae* UCG-003 was the only altered taxon with LDA score = 4.28 at the genus level (Fig. 3).

**Table 3:** Relative abundance of the dominant fecal bacterial genera (> 0.01% of total sequences) that were affected by dietary supplementation of a blend of *S. cerevisiae*-based direct-fed microbial and fermentation products

Genus (% of total sequences)	CON	PROB	SE	*P*-value
*Prevotellaceae* UCG-003	1.91	4.15	0.48	0.03
p-2534-18B5 gut group^a^	0.81	0.00	0.60	0.01
*Elusimicrobium*	0.26	0.01	0.18	0.02
*Megasphaera*	0.00	0.07	0.00	0.01
*Moheibacter*	0.08	0.00	0.05	0.04
*Comamonas*	0.06	0.00	0.04	0.01
*Dorea*	0.07	0.15	0.01	0.02
*Stenotrophomonas*	0.04	0.00	0.02	0.01
*Blautia*	0.04	0.09	0.01	0.01
*Acetitomaculum*	0.01	0.04	0.00	0.01

^a^Uncultured bacterium belonging to the indicated family

## Discussion

### Effects of PROB on plasma carbonyl-metabolome

The increased relative concentrations of plasma monosaccharides such as glucose, galactose, fructose, and glyceraldehyde in steers fed PROB diet is evidence of an improved energy status of the animals. Glucose is the primary energy source and must be sufficient in the blood stream for optimum performance of animals [21, 22]. In ruminants, glucose entry into the systemic circulation is majorly via hepatic or renal gluconeogenesis because glucose supply from direct intestinal absorption is low [22]. The major precursors for glucose synthesis in ruminants are short chain fatty acids (propionate, valerate, isobutyrate), lactate, and amino acids [21, 22]. Of all the precursors, propionate is the predominant substrate for gluconeogenesis in ruminants [22, 23]. The additive used in this study contains several microorganisms including *S. cerevisiae* and lactic acid bacteria (LAB) such as *Enterococcus lactis, Enterococcus faecium*, and *Lactobacillus casei*. *Saccharomyces cerevisiae* favors the activities of lactic acid utilizing bacteria (such as *Selenomonas ruminantium* and *Megasphaera elsdenii*) in the rumen [24, 25]. Production of lactic acid in the rumen by LAB is accompanied by the growth of lactic acid utilizing bacteria, which can metabolize lactate directly to propionate or indirectly through succinate to propionate [23, 26]. Therefore, it is reasonable to speculate that PROB supplementation altered rumen fermentation acid profile towards increased production of propionate, thereby resulting to increased glucose supply. Predictably, increased plasma concentration of glucose was accompanied by decreased plasma acetoacetate concentration. Acetoacetate is one of the three major ketone bodies (others are acetone and beta-hydroxybutyric acid) produced in the liver cells from the break down of fatty acids as a source of energy when carbohydrates are in short supply [27]. Nocek et al. [28] investigated the effect of supplementing DFM containing a combination of 2 *Enterococcus* strains (5 × 10^9^ cfu/g) and *S. cerevisiae* (2 × 10^9^ cfu/g) to lactating Holstein cows during the transition period and reported improved performance associated with higher blood levels of glucose and lower serum levels of beta-hydroxybutyric acid. Similar results were reported by Nocek and Kautz [29].

Compared to CON, greater plasma concentration of hippuric acid was observed in steers fed PROB diet. Hippuric acid is a gut-derived phenolic metabolite formed by the conjugation of benzoic acid (produced by microbial degradation of phenolic compounds) with glycine in the liver or kidney [30]. Studies in rats and humans have demonstrated that urinary and blood hippuric acid content is modulated by changes in the intestinal microbiome [31, 32]. Although rumen microbial population was not measured in the current study, increased plasma hippuric acid concentration may possibly be because PROB supplementation modulates the composition of rumen microbiota towards a more favorable production of hippuric acid. In humans, increased fasting serum hippuric acid concentration was associated with better glucose and insulin metabolism [33]. In addition, Wu et al. [34] reported increased serum hippuric acid concentration in highly efficient dairy cows. Consequently, it is reasonable to speculate that higher plasma hippuric acid in steers fed PROB diet indicates improved glucose metabolism, which corroborates increased plasma glucose concentration observed in animals fed PROB diet. Further studies are needed to infer the connection between plasma hippuric acid and energy status of animals.

Recent studies have identified tryptophan catabolism, through the kynurenine metabolic pathway, as one of the mechanisms by which the immune system modulates the balance between response to pathogens and tolerance to harmless antigens [35]. The enzyme, indoleamine 2, 3-dioxygenase that breaks down tryptophan via this pathway is induced by the immune cells during activation. Increased plasma concentration of 5-hydroxykynurenamine, an intermediate product of tryptophan catabolism [36], suggests an increased tryptophan degradation possibly due to immune cell activation. This is in line with the results of our companion study [10], which showed that PROB supplementation increased the expression of immune-related genes responsible for regulating the animal’s immune response toward intracellular and extracellular pathogens.

Phenylacetylglycine levels in the blood is primarily driven by gut microbial metabolism [37]. Formation of phenylacetylglycine by conjugation of phenylacetate has been reported to be an ammonia-lowering pathway [38]. Although previous studies have reported decreased ruminal ammonia production in animals fed *S. cerevisiae*-based additive [39–41], the biological significance of increased plasma phenylacetylglycine is not known because ruminal ammonia level was not measured in this study. Further studies are needed to determine the relationship between blood phenylacetylglycine content and ruminal ammonia levels of beef cattle.

Limited or no information is available in the literature on the significance of the other metabolites affected by PROB supplementation in this study. Some of these metabolites such as 5-oxopentanoate, isomer of (S)-2-aceto-2-hydroxybutanoate, isomer of (S)-3-methyl-2-oxopentanoic acid, 3-(4-hydroxyphenyl)pyruvate, (S)-2-aceto-2-hydroxybutanoate, (R)-3-hydroxy-3-methyl-2-oxopentanoate, and 2-dehydropantoate were positively correlated with performance parameters such as ADG and FE. More in-depth studies are needed to understand better how these metabolites are associated with improved animal performance.

The primary limitation of this study was the use of an untargeted metabolomics approach which provides relative quantification of the metabolites based on intensity values. Nonetheless, this study confirms the benefits of PROB supplementation at improving the metabolic status of beef steers. Further studies that utilize a targeted metabolomics are needed to provide absolute quantification of some of the metabolites whose functions are currently unknown. Future advances in metabolomics technology should also focus on identifying those metabolites that were putatively matched in MCID library in this study.

### Effects of PROB on fecal bacterial community

In the present study, the relative abundance of *Prevotellaceae* UCG-003 was increased in steers fed PROB diet and was the most altered taxa as revealed by LEfSe analysis. *Prevotellaceae* UCG-003 belongs to a group of bacteria, *Prevotella*. *Prevotella* plays a key role in the metabolisms of carbohydrate (such as sugar, starch and xylan) and protein [42–44]. *Prevotella* can grow effectively at low pH (< 6.0), especially in the rumen [45, 46]. Thus, the increased relative abundance of *Prevotellaceae* UCG-003 in the feces of beef steers fed PROB diet is probably an increased production of organic acids in the hindgut due to increased fermentation of carbohydrates, which is associated with reduced fecal pH [47]. This probably explains the decreased fecal pH of the beef steers reported in our companion study [10]. These results suggest the viability of the DFM used in this study in the hindgut. Previous studies have confirmed the viability of *S. cerevisiae* and lactobacilli, both of which are constituents of PROB, throughout the gastrointestinal tract of beef cattle [48, 49]. An alternative explanation for increased relative abundance of *Prevotellaceae* UCG-003 in the feces is that PROB supplementation reduced ruminal fermentation and caused a shift in the site of fermentation to the large intestine, thereby increasing substrate availability for increased growth of *Prevotellaceae* UCG-003. This scenario would be expected to reduce the performance of the animals, which is contradictory to the growth performance results reported in our companion paper [10], because the contribution of the hindgut fermentation to nutrient digestion in ruminants is substantially less than that of the rumen.

Supplementation of PROB increased the relative abundance of lactate-utilizing bacteria, such as *Dorea* and *Megasphera*. This suggests the activities of the lactate-producing bacterial content of PROB. Increased colonization of *Lactobacillus* in the hindgut could result in increased production of lactic acid, which can favor the growth of lactate-utilizing bacteria. *Blautia,* a bacterial genus that can degrade complex carbohydrates, and *Acetitomaculum*, an acetogenic bacterial genus were also increased in feces of steers fed PROB. Conversely, the relative abundance of *Elusimicrobium*, *Moheibacter*, *Stenotrophomonas, Comamonas,* and uncultured bacterium belonging to family p-2534-18B5 gut group were reduced. The relative abundance of these aforementioned bacterial genera, including *Dorea* and *Megasphera* are low (≤ 0.1%), therefore, changes in their relative abundance may not be of biological relevance. Taken together, these results indicate that PROB supplementation altered the fecal bacterial community toward increased relative abundance of *Prevotellaceae* UCG-003 and lactate-utilizing bacteria.

## Conclusion

Supplementation of PROB improved the energy status of the beef steers by increasing the relative concentrations of plasma monosaccharides such as glucose, galactose, fructose, and glyceraldehyde, as well as others (hippuric acid, phenylacetylglycine, and 5-hydroxykynurenamine) with possible health benefits. In contrast, the relative concentration of acetoacetate was reduced. The significance of some metabolites, notably 5-oxopentanoate, isomer of (S)-2-aceto-2-hydroxybutanoate, isomer of (S)-3-methyl-2-oxopentanoic acid, 3-(4-hydroxyphenyl)pyruvate, (S)-2-aceto-2-hydroxybutanoate, (R)-3-hydroxy-3-methyl-2-oxopentanoate, and 2-dehydropantoate that showed positive correlations with performance measures merits further evaluation in future studies. Supplementation of PROB alters the fecal bacterial population towards increased relative abundance of *Prevotellaceae* UCG-003 and some.

## Supplementary information

**Additional file 1:** **Table S1.** Ingredient and chemical composition of the basal diet.

**Additional file 2: Figure S1.** Mass accuracy checks of all the samples, including quality control and blank samples.

**Additional file 3:** **Table S2.** Number of peak pairs for each sample.

**Additional file 4: Table S3.** List of peak pairs detected from CIL LC-MS measurement of the samples.

**Additional file 5: Figure S2.** A. PLS-DA cross validation results (*R*^*2*^ = 0.988, Q^2^ = 0.788) and B. PLS-DA permutation test results (Empirical *P*-value = 0.009901).

**Additional file 6: Table S4.** List of altered peak pairs.

**Additional file 7: Figure S3.** A. Alpha (Shannon index; *P* = 0.34, SE = 0.45) and B. Beta (unweighted unifrac distance) fecal samples from beef steers fed no (CON) or 19 g/d of a blend of *S. cerevisiae*-based direct-fed microbials and fermentation products (PROB; *P* = 0.84, SE = 0.12).

## Data Availability

All data generated or analyzed are available from the corresponding author on request.

## Abbreviations

ADG: Average daily gain
CIL: Chemical isotope labeling
DFM: Direct-fed microbial
DnsHz: Dansylhydrazine
FC: Fold change
FE: Feed efficiency
LC-MS: Liquid chromatography mass spectrometry
LDA: Linear discriminant analysis
LEFSe: Linear discriminant analysis effect size
m/z: Mass-to-charge ratio
OTU: Operational taxonomic units
PCA: Principle component analysis
PCR: Polymerase chain reaction
PLS-DA: Partial least squares discriminant analysis
PROB: A blend of *Saccharomyces cerevisiae*-based DFM and fermentation products
QC: Quality control
TMR: Total mixed ration
