# Supplementary material for: Effects of a blend of Saccharomyces cerevisiae-based direct-fed microbial and fermentation products on plasma carbonyl-metabolome and fecal bacterial community of beef steers
Source: J Anim Sci Biotechnol. 2020 Feb 17;11:14. doi: 10.1186/s40104-019-0419-5 (PMC7025411; doi:10.1186/s40104-019-0419-5)
Supplement: Supplementary file 1 — Additional file 1: Table S1. Ingredient and chemical composition of the basal diet. [file 40104_2019_419_MOESM1_ESM.docx]

**Table S1.** Ingredient and chemical composition of the basal diet^1^

| Ingredient (%DM) |  |
| --- | --- |
| Corn silage | 79.7 |
| Dehydrated distillers grain | 9.06 |
| Soybean meal | 9.28 |
| Limestone | 0.42 |
| Deccox | 0.03 |
| Vitamin and mineral premix | 1.51 |
| Nutrient analysis | |
| Crude protein, % | 14.7 |
| Ether extract, % | 3.50 |
| Ca, % | 0.68 |
| P, % | 0.59 |
| TDN, % | 72.3 |
| Net energy of maintenance, Mcal/kg | 1.72 |
| Net energy of gain, Mcal/kg | 1.10 |

^1^Chemical composition of complete diets calculated from analysis and concentration of individual ingredients
