# Supplementary figures and images for: Effects of a blend of Saccharomyces cerevisiae-based direct-fed microbial and fermentation products on plasma carbonyl-metabolome and fecal bacterial community of beef steers
Source: J Anim Sci Biotechnol. 2020 Feb 17;11:14. doi: 10.1186/s40104-019-0419-5 (PMC7025411; doi:10.1186/s40104-019-0419-5)

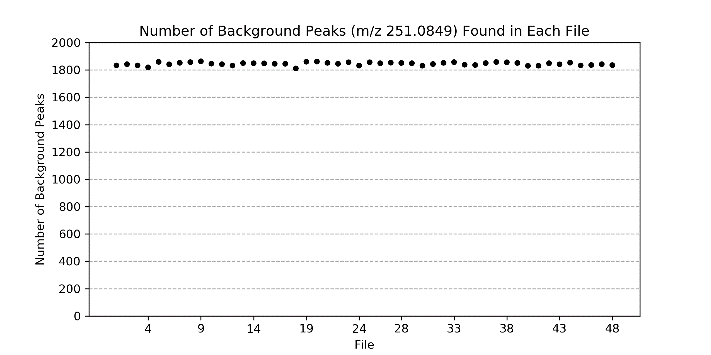

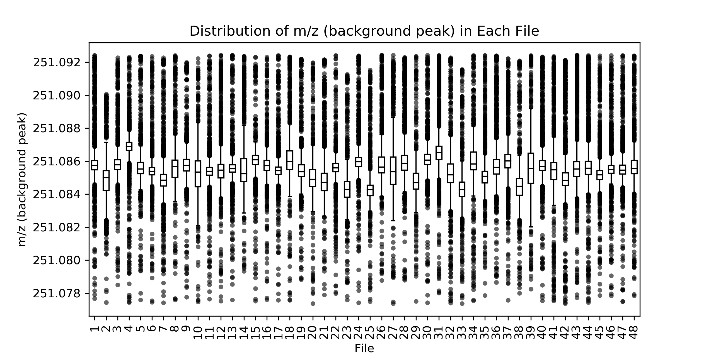


Figure S1. Mass accuracy checks of all the samples, including quality control and blank samples

Supplement: Supplementary file 2 — Additional file 2: Figure S1. Mass accuracy checks of all the samples, including quality control and blank samples. [file 40104_2019_419_MOESM2_ESM.docx]
