# Supplementary material for: Effects of a blend of Saccharomyces cerevisiae-based direct-fed microbial and fermentation products on plasma carbonyl-metabolome and fecal bacterial community of beef steers
Source: J Anim Sci Biotechnol. 2020 Feb 17;11:14. doi: 10.1186/s40104-019-0419-5 (PMC7025411; doi:10.1186/s40104-019-0419-5)
Supplement: Supplementary file 3 — Additional file 3: Table S2. Number of peak pairs for each sample. [file 40104_2019_419_MOESM3_ESM.docx]

| **Sample Name** | **Number of Peak Pairs** | **Sample Name** | **Number of Peak Pairs** |
| --- | --- | --- | --- |
| IsoMS_results_101C_31_01_4611_Spectrum.csv | 779 | IsoMS_results_22C_45_01_4625_Spectrum.csv | 764 |
| IsoMS_results_102C_60_01_4634_Spectrum.csv | 701 | IsoMS_results_23C_11_01_4598_Spectrum.csv | 789 |
| IsoMS_results_105C_32_01_4612_Spectrum.csv | 774 | IsoMS_results_24C_7_01_4594_Spectrum.csv | 795 |
| IsoMS_results_107C_47_01_4626_Spectrum.csv | 732 | IsoMS_results_26C_67_01_4639_Spectrum.csv | 777 |
| IsoMS_results_10C_70_01_4642_Spectrum.csv | 767 | IsoMS_results_29C_36_01_4617_Spectrum.csv | 745 |
| IsoMS_results_110C_22_01_4604_Spectrum.csv | 775 | IsoMS_results_30C_79_01_4646_Spectrum.csv | 737 |
| IsoMS_results_112C_43_01_4618_Spectrum.csv | 756 | IsoMS_results_41C_19_01_4601_Spectrum.csv | 767 |
| IsoMS_results_113C_56_01_4630_Spectrum.csv | 774 | IsoMS_results_51C_71_01_4643_Spectrum.csv | 786 |
| IsoMS_results_115C_59_01_4633_Spectrum.csv | 752 | IsoMS_results_60C_80_01_4647_Spectrum.csv | 768 |
| IsoMS_results_116C_35_01_4616_Spectrum.csv | 795 | IsoMS_results_67C_44_01_4619_Spectrum.csv | 751 |
| IsoMS_results_11C_34_01_4615_Spectrum.csv | 754 | IsoMS_results_76C_20_01_4602_Spectrum.csv | 777 |
| IsoMS_results_121C_8_01_4595_Spectrum.csv | 744 | IsoMS_results_79C_48_01_4627_Spectrum.csv | 739 |
| IsoMS_results_124C_24_01_4610_Spectrum.csv | 751 | IsoMS_results_80C_12_01_4600_Spectrum.csv | 742 |
| IsoMS_results_126C_46_01_4624_Spectrum.csv | 786 | IsoMS_results_81C_68_01_4640_Spectrum.csv | 780 |
| IsoMS_results_130C_58_01_4632_Spectrum.csv | 517 | IsoMS_results_83C_82_01_4649_Spectrum.csv | 792 |
| IsoMS_results_131C_72_01_4645_Spectrum.csv | 755 | IsoMS_results_84C_9_01_4596_Spectrum.csv | 757 |
| IsoMS_results_134C_23_01_4609_Spectrum.csv | 730 | IsoMS_results_87C_81_01_4648_Spectrum.csv | 758 |
| IsoMS_results_13C_10_01_4597_Spectrum.csv | 744 | IsoMS_results_90C_21_01_4603_Spectrum.csv | 746 |
| IsoMS_results_14C_69_01_4641_Spectrum.csv | 782 | IsoMS_results_QC1_4_01_4593_Spectrum.csv | 797 |
| IsoMS_results_15C_57_01_4631_Spectrum.csv | 750 | IsoMS_results_QC2_4_01_4608_Spectrum.csv | 803 |
| IsoMS_results_17C_55_01_4628_Spectrum.csv | 727 | IsoMS_results_QC3_4_01_4623_Spectrum.csv | 800 |
| IsoMS_results_18C_33_01_4613_Spectrum.csv | 714 | IsoMS_results_QC4_4_01_4638_Spectrum.csv | 772 |

**Table S2**. Number of peak pairs for each sample
