# Supplementary material for: Effects of a blend of Saccharomyces cerevisiae-based direct-fed microbial and fermentation products on plasma carbonyl-metabolome and fecal bacterial community of beef steers
Source: J Anim Sci Biotechnol. 2020 Feb 17;11:14. doi: 10.1186/s40104-019-0419-5 (PMC7025411; doi:10.1186/s40104-019-0419-5)
Supplement: Supplementary file 5 — Additional file 5: Figure S2. A. PLS-DA cross validation results (R2 = 0.988, Q2 = 0.788) and B. PLS-DA permutation test results (Empirical P-value = 0.009901). [file 40104_2019_419_MOESM5_ESM.docx]

A.


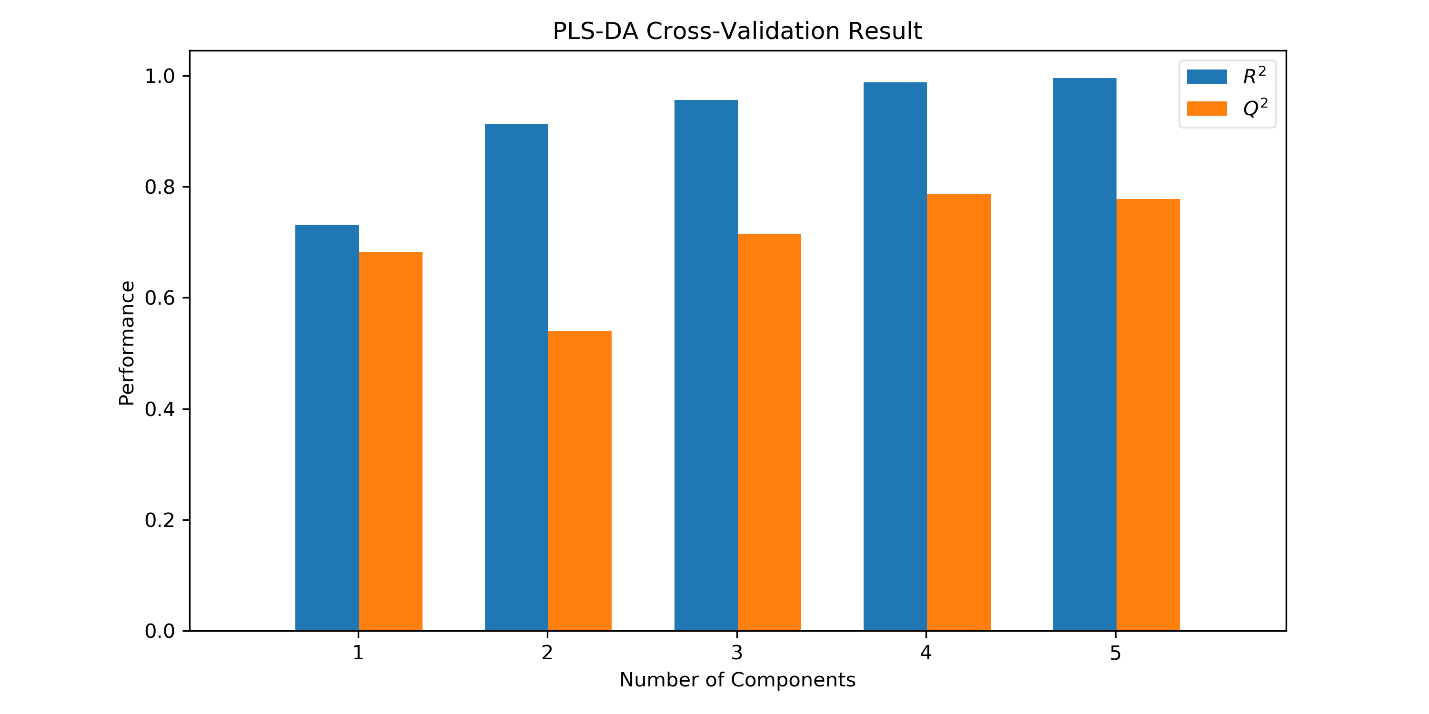


B.


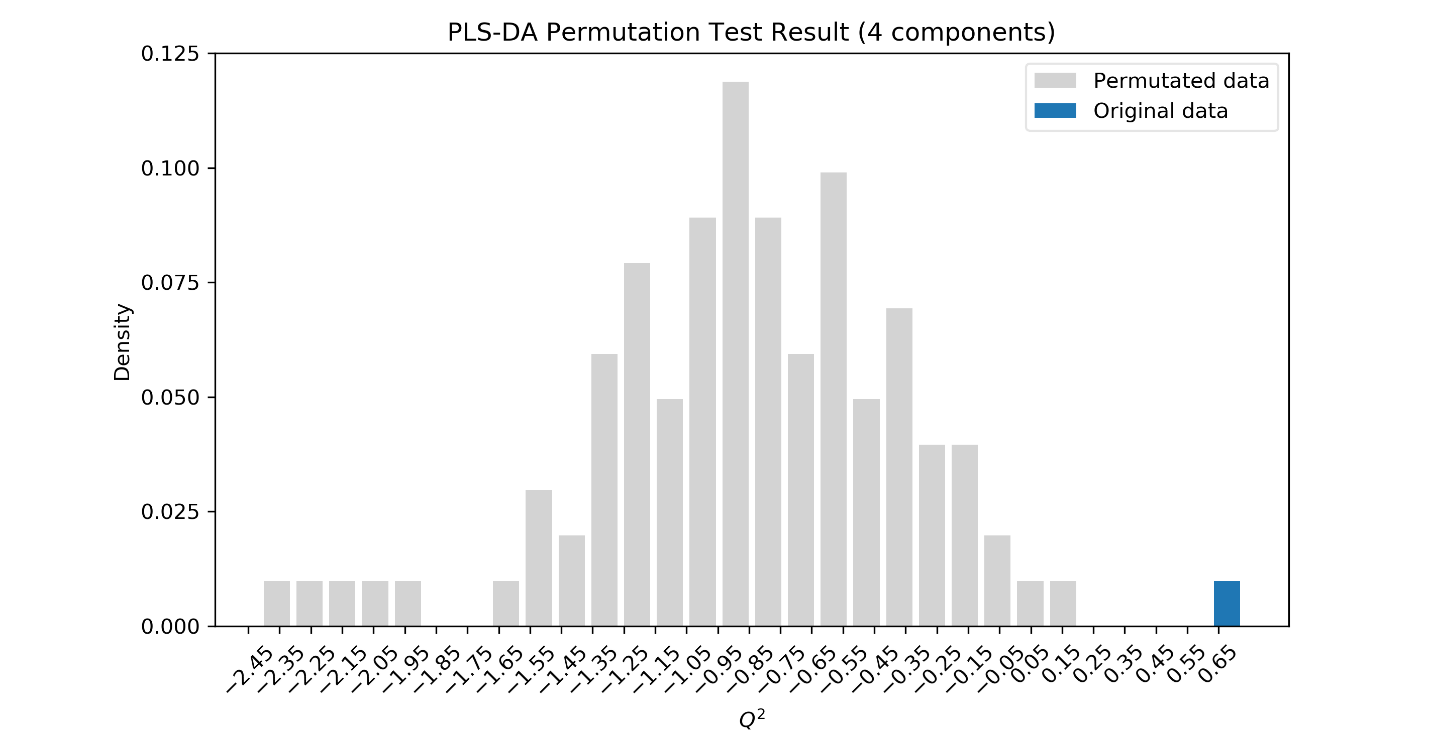


**Figure S2**. A. PLS-DA cross validation results (R^2^=0.988, Q^2^=0.788) and B. PLS-DA permutation test results (Empirical P-value = 0.009901)
