# Supplementary material for: Effects of a blend of Saccharomyces cerevisiae-based direct-fed microbial and fermentation products on plasma carbonyl-metabolome and fecal bacterial community of beef steers
Source: J Anim Sci Biotechnol. 2020 Feb 17;11:14. doi: 10.1186/s40104-019-0419-5 (PMC7025411; doi:10.1186/s40104-019-0419-5)
Supplement: Supplementary file 7 — Additional file 7: Figure S3. A. Alpha (Shannon index; P = 0.34, SE = 0.45) and B. Beta (unweighted unifrac distance) fecal samples from beef steers fed no (CON) or 19 g/d of a blend of S. cerevisiae-based direct-fed microbials and fermentation products (PROB; P = 0.84, SE = 0.12). [file 40104_2019_419_MOESM7_ESM.docx]

A.


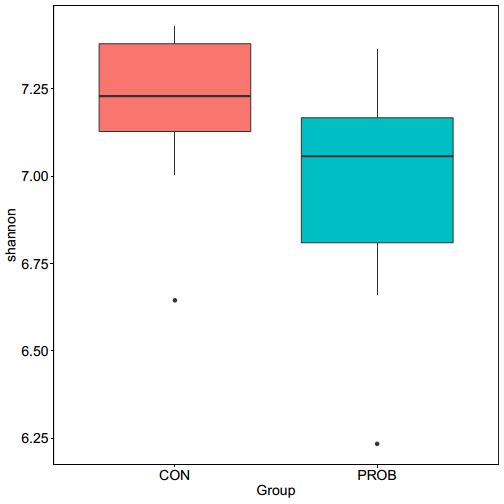


B.

**Figure S3**. (A) Alpha (Shannon index; *P* = 0.34, SE = 0.45) and (B) Beta (unweighted unifrac distance) fecal samples from beef steers fed no (CON) or 19 g/d of a blend of *S. cerevisiae*-based direct-fed microbials and fermentation products (PROB; *P* = 0.84, SE = 0.12).
